# Why make it if you can take it: review on extracellular cholesterol uptake and its importance in breast and ovarian cancers

**DOI:** 10.1186/s13046-024-03172-y

**Published:** 2024-09-06

**Authors:** Anna Røssberg Lauridsen, Aikaterini Skorda, Nuggi Ingholt Winther, Marie Lund Bay, Tuula Kallunki

**Affiliations:** 1 Cancer Invasion and Resistance, Danish Cancer Institute, Strandboulevarden 49, Copenhagen, 2100 Denmark; 2 Department of Drug Design and Pharmacology, Faculty of Health and Medical Sciences, University of Copenhagen, Copenhagen, Denmark

**Keywords:** Chemoresistant cancer, Cholesterol homeostasis, High-grade serous ovarian cancer, Lipid, Macropinocytosis, Obese, Receptor-mediated endocytosis, Statin

## Abstract

Cholesterol homeostasis is essential for healthy mammalian cells and dysregulation of cholesterol metabolism contributes to the pathogenesis of various diseases including cancer. Cancer cells are dependent on cholesterol. Malignant progression is associated with high cellular demand for cholesterol, and extracellular cholesterol uptake is often elevated in cancer cell to meet its metabolic needs. Tumors take up cholesterol from the blood stream through their vasculature. Breast cancer grows in, and ovarian cancer metastasizes into fatty tissue that provides them with an additional source of cholesterol. High levels of extracellular cholesterol are beneficial for tumors whose cancer cells master the uptake of extracellular cholesterol. In this review we concentrate on cholesterol uptake mechanisms, receptor-mediated endocytosis and macropinocytosis, and how these are utilized and manipulated by cancer cells to overcome their possible intrinsic or pharmacological limitations in cholesterol synthesis. We focus especially on the involvement of lysosomes in cholesterol uptake. Identifying the vulnerabilities of cholesterol metabolism and manipulating them could provide novel efficient therapeutic strategies for treatment of cancers that manifest dependency for extracellular cholesterol.

## Background

Changes in lipid metabolism contribute to malignant transformation by promoting the “hallmarks of cancer”, and such metabolic changes can be caused by e.g. elevated cholesterol levels and obesity [1, 2]. Obesity and dysregulated lipid homeostasis are connected to increased risk of breast cancer and correlate with worse outcomes in both breast and ovarian cancers [3–5], and increased cholesterol levels in cancer cells correlate with their increased growth, invasiveness and chemoresistance [6–8]. Since cancer cells are dependent on cholesterol, they need to learn to master the utilization and manipulation of cholesterol metabolism to promote their survival, growth, and invasiveness. In this review we discuss about the cholesterol uptake mechanisms activated and utilized by cancer cells. Understanding the various means how cancer cells fulfil their cholesterol need, including their ability to compensate decreased cholesterol synthesis with the uptake of the extracellular cholesterol, is central for the efficient targeting of cholesterol metabolism in cancer.

## Cholesterol homeostasis in healthy cells

**Fig. 1:**
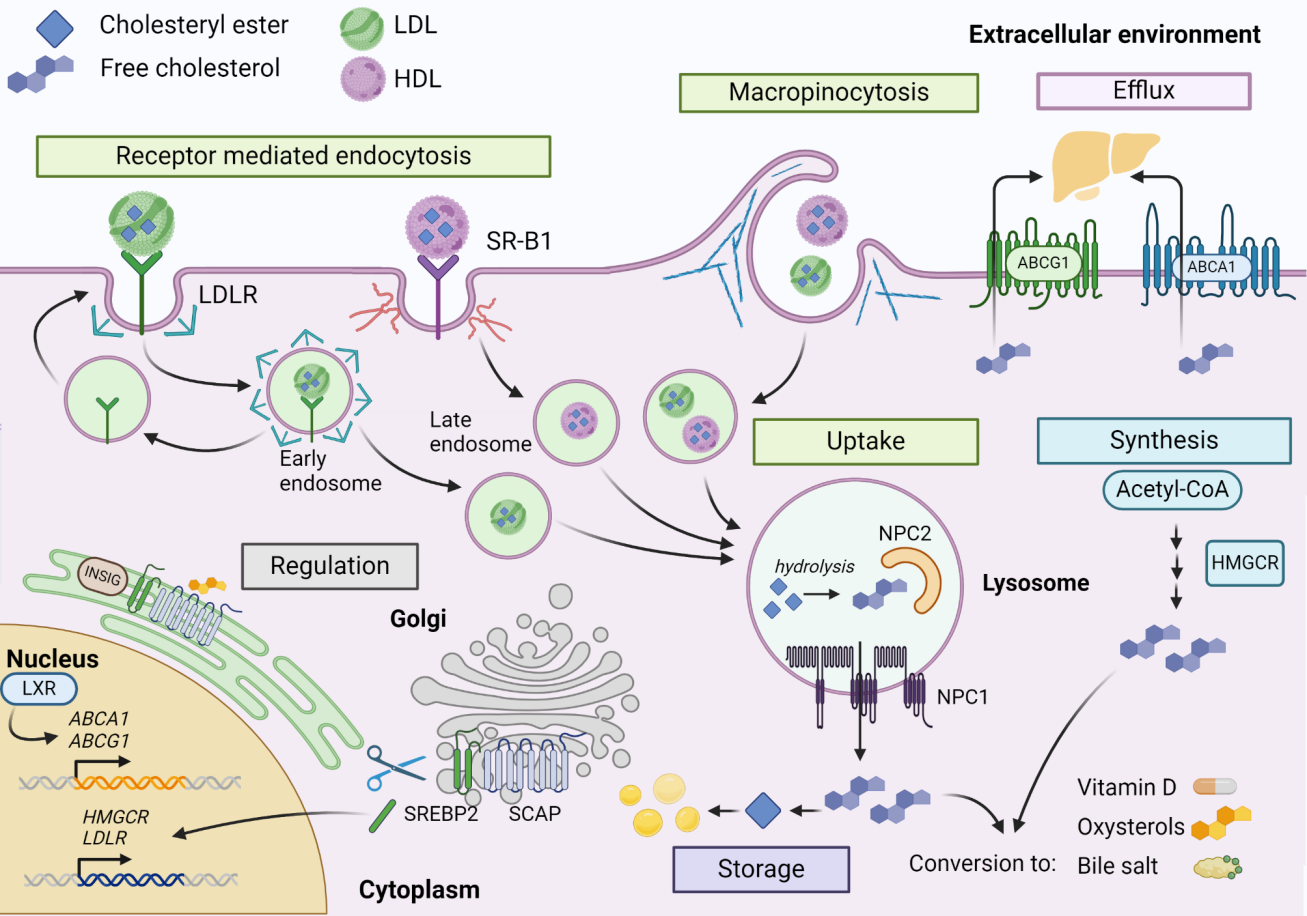
Overview of cholesterol homeostasis pathways utilized in cancer. Cells obtain cholesterol by taking it up from the extracellular environment or by *de novo* synthesis. Excess intracellular cholesterol can be transported out of cells with specific efflux mechanisms including ATP-binding cassette (ABC) transporters ABCA1 and ABCG1. Cholesterol uptake involves receptor mediated endocytosis, macropinocytosis and the cooperation of lysosomes resulting in a release of the free cholesterol from cholesteryl ester. The cholesterol levels will affect the regulatory machinery, low cholesterol levels lead to increase transcription through SREBP-2 activation and high cholesterol levels leads to the deactivation of SREBP-2, activation of LXR and the storage of cholesteryl esters in lipid droplets. ABCA1 (ATP-binding cassette transporter A1), ABCG1 (ATP Binding Cassette Subfamily G Member 1), HDL (high density lipoprotein), LDL (low density lipoprotein), LDLR (Low density lipoprotein receptor) LXR (Liver X receptor), NPC1 and NPC2 (Niemann-Pick type C protein 1 and 2), HMGCR (HMG-CoA reductase), SR-B1 (Scavenger receptor class B type 1), SREBP-2 (Sterol regulatory element-binding protein 2), SCAP (SREBP-2 cleavage activation protein), INSIG (Insulin-induced gene protein)

Cholesterol homeostasis is crucial for mammalian cells, and it is therefore tightly regulated by complex signalling networks that control its synthesis, uptake, conversion, trafficking, and efflux (Fig. 1) [9]. Cholesterol is an essential lipid, and it has multiple functions that are central for normal cellular physiology. It is the principal precursor for steroid biosynthesis and synthesis of vitamin D and oxysterols, and it is involved in proliferation signalling [9, 10]. Cholesterol forms a vital part of the plasma membrane due to its tetracyclic ring that gives the molecule a planar and rigid structure that increases plasma membrane packing and contributes critically to its stability, integrity, and fluidity. This configuration allows cholesterol to intercalate between phospholipids impacting their membrane package and interactions ensuring proper cellular function and responsiveness to environmental changes [11]. There are two main sources for cholesterol for mammalian cells: *de novo* synthesis of cholesterol in the endoplasmic reticulum and cytosol, and the uptake of extracellular cholesterol by various endocytic mechanisms via digestive system through the bloodstream or from lipolyzed adipocytes of the fatty tissues (Fig. 1) [12, 13].

### Cholesterol synthesis

Cholesterol synthesis occurs via mevalonate pathway, and it requires large amounts of energy [14, 15]. The newly synthesized cholesterol is transported to its destination, mainly to the plasma membrane, but also in a smaller extent to other cellular membranes [9]. 3-hydroxy-3-methylglutaryl coenzyme A (HMG-CoA) reductase (HMGCR) is a key enzyme of the mevalonate pathway. It mediates the irreversible conversion of HMG-CoA to mevalonate, which is a rate-limiting step in the cholesterol synthesis [16]. The sterol regulatory element-binding protein 2 (SREBP-2) is a master regulator of the mevalonate pathway gene expression [9]. It is a central transcription factor regulating the expression of the enzymes involved in cholesterol synthesis and transport. It can sense cholesterol levels with complicated mechanisms that involve interactions with other cellular components. When cholesterol levels decrease, SREBP-2 cleavage activation protein (SCAP) forms a complex with SREBP-2, and this will translocate to the Golgi apparatus where SREBP-2 is cleaved to its active form. The activated SREBP-2 enters the nucleus and initiates the transcription of its target genes. When cholesterol levels suffice, SREBP-2 is inactive and located in the endoplasmic reticulum (ER) associated with SREBP-2 cleavage activation protein (SCAP) [17]. SREBP-2 and SCAP both contain the sterol-sensing domain, a conserved core that is essential for their sterol-dependent functions [18].

A well-established way to inhibit the synthesis of cholesterol is by targeting the HMGCR with statins [19, 20]. These compounds are most efficient agents for the reduction of plasma cholesterol. Statins target hepatocytes and inhibit HMGCR by competing with its normal substrate at the active site of the enzyme. They alter the conformation of the HMGCR by binding to its active site, which prevents it from attaining a functional active structure. Inhibition of HMG-CoA leads to reduction of intracellular cholesterol in hepatocytes, which will activate the SCAP-SREBP-2 mechanism leading to an increase in the gene expression for low density lipoprotein (LDL) receptor (LDLR). The increase of hepatic LDLR will cause a reduction in the amount of circulating LDL and also LDL-cholesterol [21].

### Cellular cholesterol uptake

Cellular uptake and utilization of dietary cholesterol occurs either from the blood through the endothelial cells of the blood vessels or from the intestines through the plasma membrane of enterocytes. Cholesterol circulates in the blood as cholesteryl esters which are packed as LDLs or high-density lipoproteins (HDL). Normal mammalian cells, but mainly hepatocytes, adipocytes, macrophages, intestinal epithelial cells as well as endothelial and smooth muscle cells lining the blood vessels take up cholesterol from the blood by receptor-mediated endocytosis requiring of LDL binding to LDLR. The HDL receptor, the Scavenger receptor class B type 1 (SR-B1; SCARB1), binds HDL on the surface of the receiving cell to transfer the HDL bound cholesteryl esters to hepatocytes.

**Fig. 2:**
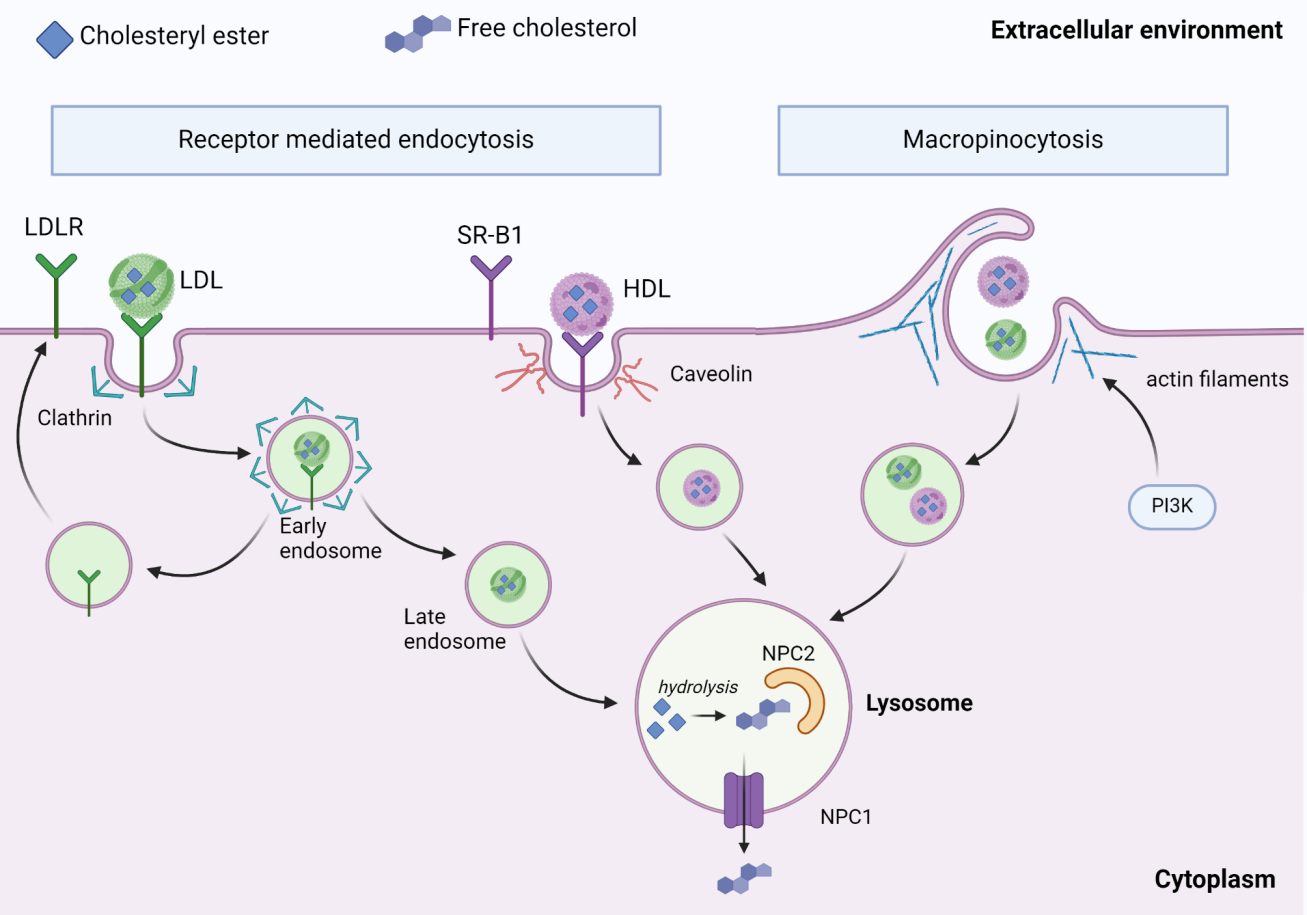
Overview of extracellular cholesterol uptake pathways utilized in cancer cells. Cells can efficiently take up and utilize extracellular cholesterol mainly by two different mechanisms: a receptor-mediated endocytosis involving LDLR or SR-B1, or by macropinocytosis, a receptor independent internalization mechanism which involves actin filament reorganization under the plasma membrane. Cholesteryl esters that are taken up from the extracellular environment are hydrolysed to cholesterol in lysosomes and transferred to NPC2 and then outside of lysosome through NPC1. All these pathways are often upregulated in cancer

When the ligand-receptor complex is formed, consisting of the lipoprotein bound cholesteryl ester in either LDL or HDL and their corresponding receptors, this complex will be endocytosed and internalized into early endosomes (Fig. 2). Due to the low pH of the early endosomes, lipoprotein dissociates from its receptor while the early endosome matures into a late endosome [13]. Meanwhile, in the case of LDL, the LDLR is mostly recycled back to the plasma membrane and the LDL remains in the maturing endosomal system. Here it will be transported through several intermediate endosomal vesicles which are maturated by their fusion and fission until reaching the lysosomes [22, 23]. In the lysosomes the LDL particles are degraded, and cholesteryl ester will be hydrolysed to release the free cholesterol, which can either be trafficked to the plasma membrane by sterol transfer proteins or be converted to cholesteryl ester by sterol O-acetyltransferase (SOAT1 or ACAT1) and stored in lipid droplets in cytosol or be catalysed into oxysterols, bile acids or steroid hormones.

### Cholesterol efflux and storage

**Fig. 3:**
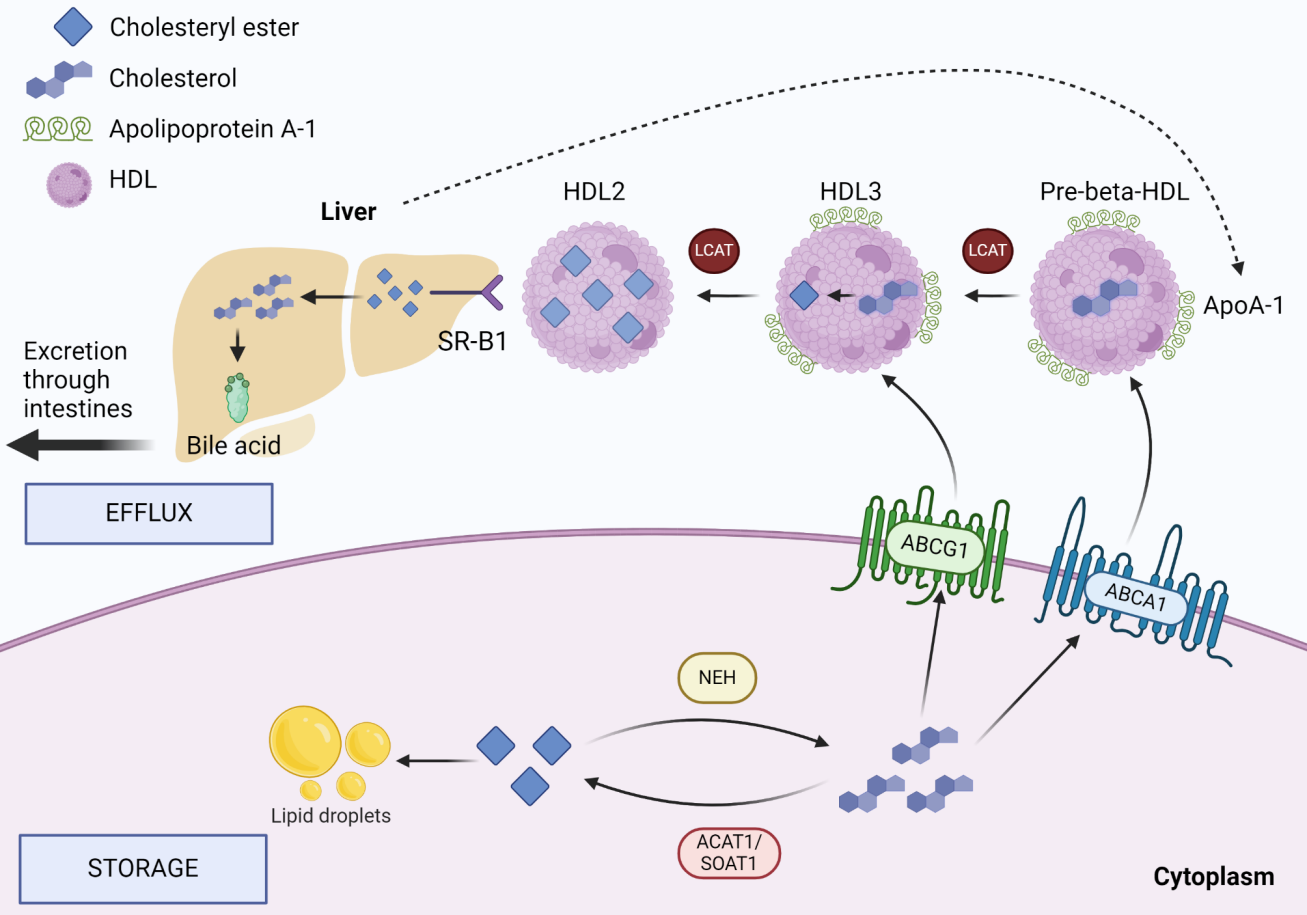
Overview of the cholesterol efflux and storage system. Excess intracellular cholesterol is stored as lipid droplets in cytosol or transported out of the cell via ABC transporter here represented by ABCA1 and ABCG1. Lecithin-Cholesterol Acyltransferase (LCAT) esterifies free cholesterol into cholesteryl ester on the surface of the HDL forming HDL2 and HDL3 which are modified forms of HDL with decreasing amount of cholesterol on their surface. HDL particles are transported to the liver for excretion through the intestine. Cholesterol Acyltransferase 1 (ACAT1/SOAT) esterifies intracellular free cholesterol to be stored in lipid droplets. When stored cholesterol is needed, Neutral Lipid Ester Hydrolase (NEH) releases stored lipids and cholesterol for cellular use

In addition to cholesterol synthesis and uptake mechanisms, specific cholesterol efflux and storage systems are needed for cells to maintain their cholesterol homeostasis [24, 25] (Fig. 3). Excess intracellular cholesterol is highly toxic for cells and its level must be controlled to maintain cellular viability. In addition to generating damaging oxidative molecules, excess cholesterol will lead to reduction of membrane fluidity and disrupt signalling from lipid rafts, which are cholesterol and sphingolipid rich structures in the plasma membrane that regulate the assembly and functioning of numerous cell signalling pathways and contribute to their proper function [9].

Excess intracellular cholesterol can be stored as cholesteryl esters in the lipid droplets in cytosol as cholesteryl esters or excreted to the bloodstream by ATP-binding cassette (ABC) family transporters such as ABCA1 or ABCG1, which can interact with HDL and deliver the cholesterol to it [13, 22, 26–28] (Fig. 3). In normal physiology, SR-B1 has an essential role in the reverse cholesterol transport pathway, where it facilitates the removal of excess body cholesterol by allowing the excretion of cholesterol as part of HDL molecules from peripheral cells to the liver and gallbladder [29]. Lipid droplets are mostly located in cells and tissues that are involved in lipid metabolism such as hepatocytes and in the visceral fat of the adipose tissue. Interestingly the ABC transporters are often upregulated in chemoresistant cancers, since they efficiently excrete chemotherapeutic drugs such as doxorubicin, taxane- and platinum-based drugs from cancer cells.

### Regulatory mechanisms

**Fig. 4:**
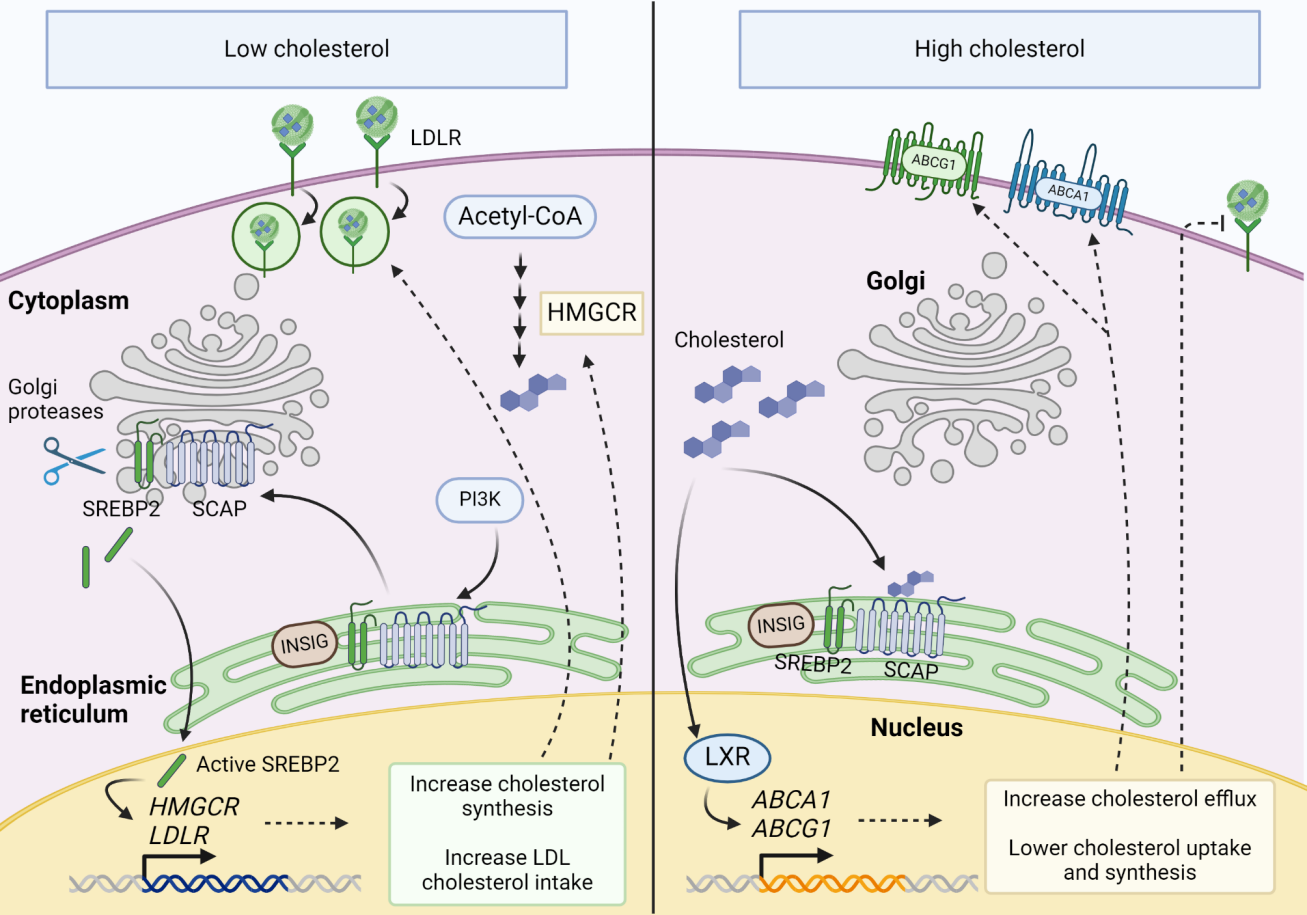
Cellular mechanisms for sensing and responding to altered cholesterol levels. Left: Low cholesterol levels lead to upregulation of cholesterol synthesis and uptake. SCAP senses low cholesterol levels and takes SREBP2 from Golgi to the endoplasmic reticulum (ER) for activation, after which SREBP2 enters nucleus and activates target gene transcription. Right: High cholesterol levels lead to increased cholesterol efflux and decreased cholesterol uptake and synthesis. Here also SCAP acts as a cholesterol sensor. Cholesterol binds to SCAP causing it to retain the SREBP2 in the ER to prevent its transport to the nucleus. INSIG proteins play a central role in this process by regulating the SREBP2-SCAP complex assembly

Various regulatory mechanisms finetune cholesterol metabolism to maintain cellular viability [9]. Several proteins are needed to regulate cellular cholesterol homeostasis (Fig. 4). These include LDLR, SR-B1, HMGCR, SREBP-2, SCAP, NPC1, NPC2 and Liver X receptor (LXR) among others [18, 30].

Increase in intracellular cholesterol content and storage depends on activation of PI3K-AKT-mediated activation of SREBP pathway [31] which induces *de novo* synthesis of sterol or preserves the LDLR-mediated cellular uptake [32, 33]. In addition to a master regulator of cholesterol metabolism, SREBP-2, cholesterol levels are influenced to some extent by other transcription factors such as LXR. High cholesterol levels activate LXRs, resulting in the inhibition of cholesterol synthesis, activation of cholesterol efflux via increased expression of ATP binding cassette transporters and reduced cholesterol uptake [9] (Fig. 4).

## Lysosomes in cholesterol uptake and utilization of extracellular cholesterol

Lysosomes are acidic organelles, whose main function is to break down macromolecules and recycle their breakdown products. Lysosomes are composed of an acidic lumen surrounded by lipid bilayer membrane. The acid lumen contains hydrolytic enzymes, including nucleases, proteases, phosphatases, lipases, and sulfatases, which are needed in breaking down different type of lysosomal cargo [34]. In addition to the degradation and recycling of worn-out cellular components, lysosomes are involved in complex biological functions most of them relying on their digestive feature. These functions include regulation of cellular signalling, metabolic activity, plasma membrane repair and remodelling of the extracellular matrix [23].

Lysosomes receive their substrates through the endocytic pathways or via autophagy. When endosomes fuse to form late endosomes and lysosomes, cholesterol has been released from its lipoprotein carriers HDL or LDL and it will be in the form of cholesteryl ester. The cholesteryl ester is hydrolysed to free cholesterol in the lysosomes with the help of lysosomal acid lipase (LAL) [35]. From here Niemann-Pick disease type C1 and C2 (NPC1 and NPC2), which are cholesterol transport proteins at the lysosomal membrane and lysosomal lumen, respectively, will be responsible for the transport of the free cholesterol to the cytosol.

### Lysosomes and cholesterol homeostasis

Lysosomes enable cellular accessibility of extracellular cholesterol and cholesteryl esters [35]. The late endosomal and lysosomal membrane protein, cholesterol transporter NPC1, plays a key role in this. It consists of 13 transmembrane domains, four small and three large luminal loops, six small cytoplasmic loops and lastly, a cytoplasmic tail [36]. Free cholesterol binds in the lysosomal lumen to NPC2, which then transfers cholesterol to the sterol-binding pocket of NPC1 [35]. Cholesterol is then further transported to the sterol sensing domain in the third membrane helix of NPC1, from where it is finally transferred across the lysosomal membrane and out in the cytosol [16].

The most abundant group of the lysosomal membrane proteins are the lysosome-associated membrane proteins 1 and 2 (LAMP-1 and LAMP-2) and the CD36 superfamily member, lysosomal integral membrane protein-2 (LIMP-2 or SCARB2) [22]. Of these LAMP-2 and LIMP-2 are directly involved in cholesterol homeostasis and can bind cholesterol. While LIMP-2 acts as a cholesterol carrier, LAMP-2 is involved in its storage [35]. NPC2 can also deliver free cholesterol to LAMP-2, whose luminal domain interacts with NPC1, thus serving as a storage source of free cholesterol prior to NPC1-mediated efflux [35]. This additional function of LAMP proteins apart from their best established role in maintaining lysosomal membrane stability, underlie the dominance of advanced lysosome-sterol mediated interactions and signalling effectors [22].

Inactivating mutations in *NPC1* or *NPC2* will cause accumulation of cholesterol in the lysosomal lumen and result in disruption in cholesterol homeostasis causing the fatal neurological disease Niemann-Pick Type C (NPC) disease [37, 38]. The export of cholesterol from the lysosomal lumen by NPC1 is essential for the regulation of mTORC1 signalling, thus mutations resulting in the inactivation of NPC1 will not only result in the accumulation of cholesterol but also as the hyperactivation of mTORC1, whose activation is often associated with cancer [39].

### Inhibition of cholesterol transport through lysosomes

Inhibition of NPC1 with its chemical inhibitor U18666A can mimic the loss of function of the NPC1 observed in Niemann-Pick Type C diseases. U18666A is a cationic sterol that crosslinks directly to NPC1, which causes its sterol sensitive domain to change configuration and therefore inhibiting the passage of cholesterol in the cells [28]. U18666A can thus be used to investigate the effects of blocking the cholesterol trafficking through the lysosomal pathway in cells [40]. U18666A is a lysosomotropic compound, a compound that accumulates in lysosomes it can directly inhibit NPC1 and thus the intracellular trafficking of cholesterol [41]. This will lead to accumulation of cholesterol inside lysosomes which can result in prevention of tumor growth and invasion in some cancers [26, 41]. Treatment with U18666A will result in a mimicking of loss-of-function mutations of NPC1, leading to an imbalance in the cholesterol level in the cell, thereby affecting membrane trafficking, communication between organelles, cellular homeostasis and inducing death [40, 42].

Itraconazol is another compound that can block cholesterol trafficking in cells. It is an anti-fungal agent that has been repurposed for cancer treatment due to its ability to reverse chemoresistance and to inhibit hedgehog and mTOR signalling, angiogenesis and autophagy [43, 44]. It has similar effect as U18666A on blocking the cholesterol transport out of the lysosomes, which is mediated through its binding to NPC1 in the same binding pocket on the sterol sensing domain as U18666A [45].

### Lysosomes in cancer

Lysosomes play a central role in cancer, as they can regulate cancer cell proliferation by manipulating growth factor signalling and by providing nutrients [34]. In breast cancer lysosomes have a key role in invasion mediated by the oncogenic transcription factor Myeloid Zing Finger 1 (MZF1) [46]. Constitutively active, N-terminally truncated and standard therapy resistant ErbB2/HER2 induces invasion of breast cancer cells through its downstream signalling network, which upon activation lead to the phosphorylation and activation of MZF1 at Ser 27. Activated MZF1 induces expression of the lysosomal cysteine cathepsins B and L [47, 48] and several other cancer relevant genes [46], and interestingly also the expression of cholesterol transporter NPC1 leading to metabolic switch from cholesterol synthesis to cholesterol uptake and linking invasiveness to cholesterol uptake [7]. Lysosomal cysteine cathepsins B and L are often overexpressed in aggressive tumors, and upon their transfer to the invadosomes and following secretion to the extracellular space, they can participate in the degradation of the extracellular matrix [46, 48]. This in turn increases cellular motility, invasion, and angiogenesis [49, 50]. This is likely to be partially fuelled by the energy released when switching from cholesterol synthesis to macropinocytosis-mediated uptake [7].

## Cholesterol in cancer cells

Malignant progression associates with higher cellular demand for cholesterol. Cholesterol is necessary for the formation of the plasma membrane microdomains known as lipid rafts, which organize the signalling molecules involved in cancer development and progression. Oncogenic signalling pathways that are modified by cholesterol are activated in majority of cancers, such as PI3K-Akt-mTOR, Ras-Raf-MAPK and Hedgehog pathways [33, 51].

Cells have a feedback mechanism in which free intracellular cholesterol inhibits HMGCR activity and the uptake of LDL through LDLR. As cancer cells require high levels of cholesterol, elevated LDL uptake is often seen in cancers, as well as upregulation of LDLR, NPC1, SREBP-2, and the enzymes involved in the mevalonate pathway, resulting in an increased amount of cholesterol in the cells [10, 26]. Cholesterol availability supports cell proliferation and membrane biogenesis since rapidly proliferating cancer cells have continuous need for plasma membrane components [52]. Consequently, clinical, and experimental studies suggest that cancer progression and tumorigenesis can depend on cholesterol deregulation [53, 54]. Different cancer types accumulate cholesterol through different pathways to meet their high-proliferative potential and to escape cell death. Cholesterol levels may be used as a biomarker for cancer, and it has been suggested as a pharmacological target to suppress the progression of cancer by cholesterol-lowering drugs to alleviate the clinical outcome [55]. In principle, preclinical studies often seem to correlate the overexpression of genes mediating cholesterol metabolism and transport with progression of the disease. Controversial statements arise from epidemiological studies [55, 56], reflecting the fact that the high serum LDL levels do not necessarily correlate with cholesterol uptake levels in the tumors since cholesterol uptake is regulated by tumors and their individual cancer cells.

## Cholesterol uptake and storage mechanisms in cancer cells

Extracellular cholesterol uptake is often increased in cancer cells [7, 57–59]. Many epithelial cancers, like breast and ovarian cancers, grow in the vicinity or invade into adipose tissue and several studies report on crosstalk between fat tissue and tumors. Lipid rich environment provides survival and growth advantage and enhances migration of cancer cells [60, 61]. Extracellular cholesterol is taken into the cancer cells via endocytosis and there are three major types of endocytic processes that are connected to cancer: phagocytosis, macropinocytosis and receptor-mediated endocytosis. These three endocytic processes can be distinguished by the size of the endocytosed vesicles, the characteristics of what is taken in, and the endocytic machinery involved [62]. While macropinocytosis and receptor-mediated endocytosis are processes that cancer cells utilize directly, cancer related phagocytosis is a mechanism used by macrophages to eliminate cancer cells and a part of the antitumor immune response [63], and it will not be discussed further in this review.

### Receptor-mediated endocytosis in cancer

Cancer cells use receptor-mediated endocytosis for the selective internalization of specific cell surface proteins. Cholesterol is classically taken into cancer cells as HDL or LDL lipoprotein particles via SR-B1 (receptor for HDL) or LDL receptor, which bind HDL and LDL cholesterol respectively. After receptor-cholesterol internalization, a series of cellular sorting events will determine if the internalized proteins will be processed in lysosomes or recycled back to the plasma membrane. Receptor mediated endocytosis can be further divided into clathrin-dependent endocytosis or clathrin-independent endocytosis, of which the clathrin-dependent endocytosis is involved in the uptake of LDL and HDL cholesterol [64]. Figure 2 shows a schematic representation of receptor mediated endocytosis and macropinocytosis.

#### LDL receptor and cancer

ER-negative breast cancer cell lines MDA-MB-231 and MDA-MB-436 exhibit higher proliferation rates after exogenous LDL exposure, which further stimulates cholesterol uptake and storage machinery. This does not however apply for ER-positive breast cancer cell line MCF7, T47D and ZR-75, which may be explained by the intrinsic characteristics of the molecular statuses of different breast cancer cell lines [60, 65]. Similarly, exposure to LDL-rich medium enhances cell viability and proliferation leading to larger and more aggressive tumors of ER-negative cells in comparison to lipid depleted medium [61]. Supportively the xenografts of these ER-negative 4T1 and MDA-MB-231 breast cancer cells grow larger and are more metastatic with high cholesterol diet. Loss of adhesive cellular features and increased lung metastasis potential is introduced specifically after LDL addition, while HDL exposure did not confer any effect on these tumor subtypes and stages in this experiment [60].

Generalizing the idea, obesity and dyslipidaemia may affect breast cancer development as characterized by tumor onset and growth with exacerbated aggressiveness and distant tissue metastasis in in vivo xenograft mouse models [61, 66, 67]. Alike, a cholesterol-enriched, western-type diet, triggered tumor incidence and advanced its histological grade in Transgenic Adenocarcinoma of the Mouse Prostate (TRAMP) prostate cancer mouse model [68].

#### HDL receptor SR-B1 and cancer

SR-B1 facilitates the uptake of cholesteryl esters from circulating HDL. SR-B1 is consistently overexpressed in most cancer cells [69]. Furthermore, in vitro analysis has shown that for example breast cancer cells exhibit increased proliferation and migration in the presence of HDL [70]. SR-B1 mediates selective transfer of cholesteryl ester from HDL complex to cells. Signalling functions of HDL are dependent on HDL binding to SR-B1 leading to the activation of the MAPK and PI3K-Akt signalling pathways [9]. Abnormal cholesteryl ester accumulation in breast cancer is often accompanied with enhanced expression of SR-B1 [71].

Expression of SR-B1 receptor, *SCARB1* mRNA, and consequently SR-B1 protein levels are induced by hypercholesterolemia in mouse models of breast cancer [67] and are connected to aggressiveness of cancer. Supportively, inhibition of the SR-B1 receptor via introduction of its function-disabling mutant form in MCF7 breast cancer cells inhibits their proliferation [72], supporting an earlier study where HDL as a media supplement augments proliferation in ER-positive breast cancer cells [65].

### Macropinocytosis

Macropinocytosis is a non-selective liquid-phase endocytosis process where extracellular fluid and its content are internalised into cells through a mechanism where actin-rich structures rise up from the cell surface and collapse back down forming a macropinosome [62, 73]. The result of macropinocytosis is a massive internalisation of extracellular fluid and associated solute molecules, nutrients, antigens, and lipids including cholesterol. Macropinocytosis can be considered as a more efficient internalisation over other endocytic pathways due to its robustness and its independency of specific cell surface receptor.

Macropinocytosis is activated in many cancer cell types upon extracellular stimulus such as exposure to phorbol esters, cytokines, and growth factors. Macropinocytosis is initiated by changes in the dynamics of cortical actin and is often associated with oncogene activation and regulated by intracellular proteins and their signalling that controls actin polymerization [74]. Macropinosomes are self-organized structures, heterogeneous in size and lacking an apparent coat structure. Generally, macropinosomes are recognized as being larger than 0.2 μm in diameter and capable for reaching a diameter size of up to 5 μm [75, 76].

Macropinocytosis in cancer is often activated by Ras pathway and/or the stimulation of EGF receptor (EGFR), and it includes activation of the MAPK signaling pathway [77, 78]. An additional signalling pathway known to regulate macropinocytosis is the PI3K pathway [62], which is one of the central signalling pathways activated in various malignant tumors. PI3Ks regulate the activation of macropinocytosis though phosphorylation and activation of phosphatidylinositol [3–5]-triphosphate (PIP3) and macropinosome formation involves PIP3 association with actin-rich membrane ruffles in a process that utilize cortical actin which lies just underneath the plasma membrane [74]. There are several types of PI3Ks involved in macropinocytosis: PI3K1 and 2 are associated with membrane ruffles while PI3K4 is involved in the conversion of ruffles into vesicles [79].

A recent study introduces macropinocytosis as an alternative method for cholesterol uptake in breast cancer cells [7]. The expression of constitutively active, standard immunotherapy treatment (trastuzumab and pertuzumab) resistant, truncated p95-ErbB2 in breast cancer cells activates macropinocytosis leading to increased uptake of extracellular cholesterol. The increase in the cholesterol uptake is connected to the increase in the expression of NPC1, suggesting that the upregulation of macropinocytosis and NPC1 expression allows cells to utilize extracellular cholesterol. Moreover metabolic shift from cholesterol synthesis to its uptake induces invasiveness in a manner that requires NPC1 [7]. The advantages of macropinocytic uptake of cholesterol for cancer is obvious: cells can shut off their energy consuming cholesterol synthesis and use the energy released for other cellular processes such and migration and invasion.

### Cholesterol storage in cancer

PI3K-Akt pathway is one of the most common survival pathways activated in cancer and a target of anti-cancer therapeutics. Increase in intracellular cholesterol content and storage depends on activation of PI3K-Akt-mediated stimulation of SREBP pathway [31], which induces *de novo* synthesis of sterol or preserves the LDLR-mediated cellular uptake [32, 33]. One characteristic example for this is the consequent cancer aggressiveness and bone metastasis of prostate cancer due to genetic loss of tumor suppressor PTEN and the Akt-induced aberrant accumulation of esterified cholesterol in lipid droplets [53, 80]. On the contrary, inhibition of cholesterol storage results in suppressed tumor growth of mouse prostate cancer xenografts [53], while pharmacologic disruption of PI3K-SREBP-dependent LDLR activation induces glioblastoma tumor cell death [32].

Aberrant intracellular storage of cholesteryl esters as lipid droplets correlates with a variety of other aggressive cancer types such as leukemia [81], glioma [82] and pancreatic [83] cancers, where restraining esterification represses cell proliferation, induces apoptosis, or suppresses tumor growth, respectively. Interestingly, in pancreatic cancer poor patient prognosis was connected to ACAT1 expression [83]. Abrogation of cholesteryl ester accumulation via enzymatic inhibition or depletion of ACAT1 was identified as a potent therapeutic strategy to intercept cancer progression and tumor metastasis in orthotropic mouse model of pancreatic cancer. Here cholesteryl esters are thought to assist by mediating constant signalling for sustained cholesterol metabolic activity and membrane biogenesis, while maintaining a non-toxic cellular environment containing low levels of free cholesterol [83].

## Statins and cancer

Cholesterol levels have been suggested as a potential biomarker for cancer, but also as a pharmacological target to suppress the progression of the disease [84]. Statins are the most widely subscribed cholesterol-lowering drugs. Several studies exist and are on the way to investigate the possible anti-cancer effect of statins. It is postulated that statins could have two different ways of exhibiting an anti-cancer effect. The first is by inhibition of HMGCR, thereby lowering the level of cholesterol in cancer cells, which can result in an inhibition of tumor growth, as cells require cholesterol for establishing the membranes for their daughter cells. Another way for statins to exhibit anti-cancer effect could be by preventing the activation of several oncogenic proteins, such as GTPases. GTPases are activated by metabolites formed from the mevalonate pathway, thus by inhibiting HMGCR, statins inhibit not only the synthesis of cholesterol, but also the other metabolites produced by the pathway [85–87]. Studies have found for example that simvastatin inhibits the proliferation, invasion, and migration of the ovarian cancer cell lines OVCAR3 and SKOV3, thereby possibly exhibiting anti-metastatic effects also in ovarian cancer in vivo [88, 89].

A retrospective population-based study for the association between statin use and improved survival in ovarian cancer patients was performed on 5416 patients diagnosed with invasive epithelial ovarian cancer between 2004 and 2012. According to the study epithelial ovarian cancer patients who were prescribed statins after being diagnosed, had a significant reduction in the ovarian cancer associated mortality when comparing to the patients who did not use statins [85]. There are clinical trials which have demonstrated that some ovarian cancer patients benefit from statins alone and especially when combined with chemotherapeutic agents [90]. However, there are also studies which have shown little, or no effect of statin use in ovarian cancer patients [84]. Several studies have been conducted investigating statins in in vitro models, some showing an effect of statins on cancer cell death, proliferation, and migration, while others have shown no or little effect [84]. This all demonstrating considerable variation in the cellular response to statins.

Likewise, a large population-based cohort study among close to 15,000 women in New Zealand showed a statistically significant decreased risk of breast cancer-specific death after post-diagnosed use of statins [91]. There were suggestions of effect modification across subgroups, so that statins were more protective for ER + cancers, in postmenopausal women, in late-stage patients, as well as in ‘prevalent’ statin users. In a Danish study with over 18,000 breast cancer survivors indicated that statin users have reduced rate of recurrence compared to non-users [92]. Similar allegations result from a systematic review from a total of 15 individual studies from PubMed. According to a systematic review and meta-analysis containing several studies and over 150,000 patients with breast cancer, post-diagnosis statin use decreased the risk of breast cancer recurrence and breast cancer mortality [93]. Currently 52 clinical trials on breast cancer and statins and 14 on ovarian cancer and statins are listed in the global clinical trials website clinicaltrials.gov.

## Cholesterol and breast cancer

Cholesterol is a precursor of steroid hormones including the female hormones progesterone and estrogen. In breast cancer cholesterol abundance relates to cancer progression, invasion, and metastatic ability. Most steroid-receptor positive breast cancers respond well to the hormone targeting therapies during the early stages, but often develop resistance and become independent of hormones [94]. PI3K-AKT-mediated overactivation of SREBP and cholesterol accumulation has been suggested as a causative effect for the endocrine therapy resistance in hormone responsive breast cancers [31].

Deregulation of genes involved in cellular distribution of cholesterol can contribute to cancer aggressiveness, predominantly via changes in plasma membrane lipid-raft association. For example, the StAR-related lipid transfer protein 3 (*STARD3)* gene overexpression is evident in a variety of breast carcinomas [95] and especially in connection to HER2 amplification [96], exhibiting decreased adhesiveness of breast cancer cells as well as increased metastasis, and poor patient prognosis. Moreover, STARD3 can mediate increase in the plasma membrane cholesterol content and promote lipid raft dependent signalling of lipid anchored Src kinase that in turn, modulates cell adhesion by induction of focal adhesions via activation of the Focal adhesion kinase (FAK) in HER2-negative breast cancer cells [96].

Metastasis potential has been linked with stemness, and stemness is associated with cholesterol metabolism in various carcinomas [97, 98]. Specifically, elevated cholesterol synthesis gene expression was found prevalent in breast cancer stem-cell tumorspheres and inhibition of the cholesterol synthesis pathway by statin hindered the sphere formation in vitro [99]. On the other hand, atorvastatin-treatment induced LDLR expression in breast cancer tumors and MCF-7 breast cancer cell line [100]. Upregulation correlated with increased proliferation measured by Ki67 protein levels indicating a possible connection between atorvastatin-induced LDLR upregulation and cancer aggressiveness as well as the ability of statins to target breast cancer cells. Likewise, inhibition of extracellular cholesterol uptake by macropinocytosis in breast cancer spheroids harbouring HER2 activation inhibited their invasiveness, which could be restored by extracellular LDL [7].

**Fig. 5:**
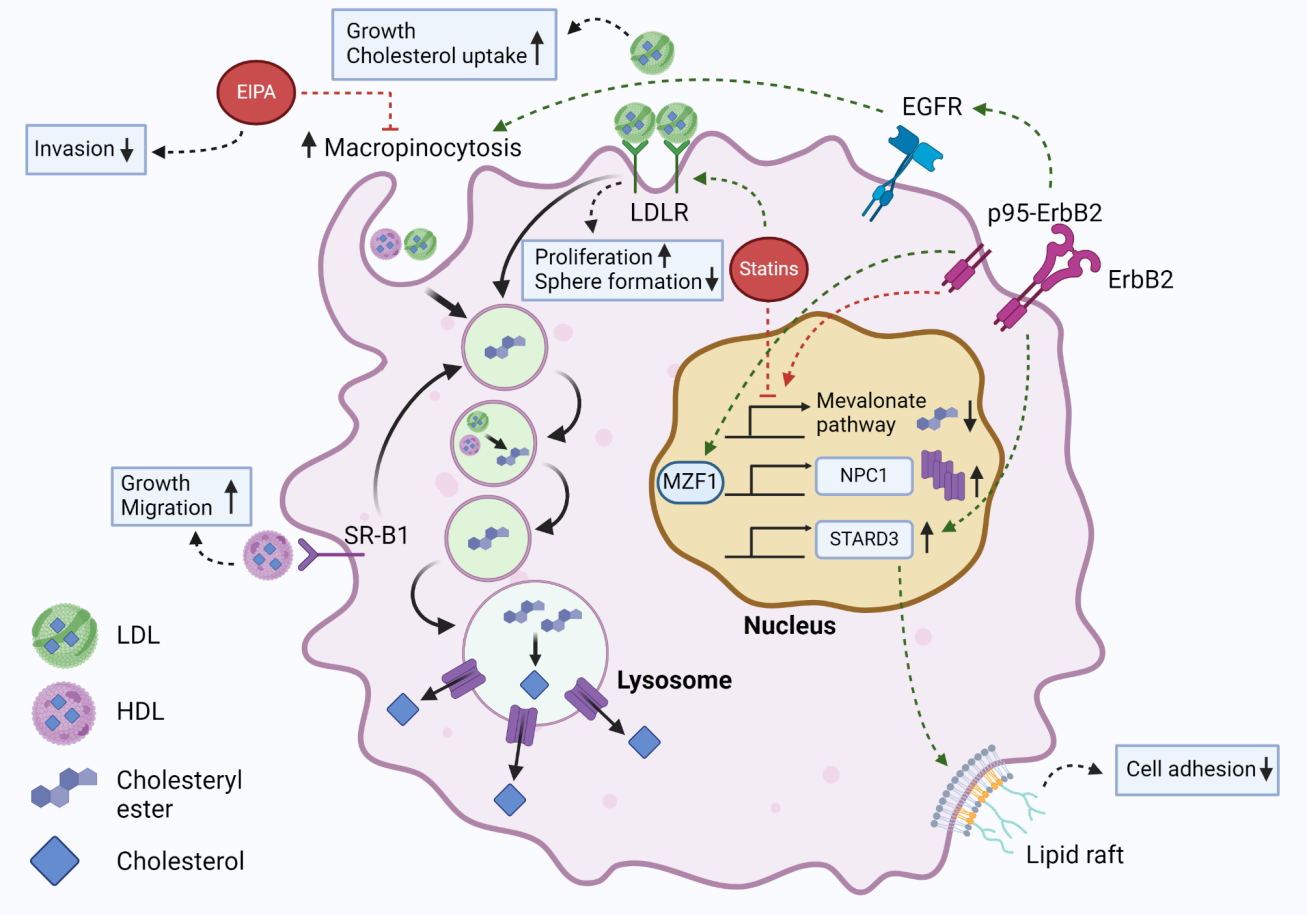
Illustration of cholesterol uptake mechanisms in breast cancer. The illustration highlights central molecular mechanisms that confer altered cholesterol metabolism in breast cancer cells. The figure includes mechanisms found in ER-negative, ER-positive and triple negative breast cancer. Abbreviations: LDL (low density lipoprotein), HDL (High density lipoprotein), NPC1 (Niemann-Pick disease, type C1), STARD3 (StAR-related lipid transfer protein 3), MZF1 (Myeloid zinc finger 1), EGFR (Epidermal growth factor receptor), ErbB2 (erythroblastic oncogene B 2)

Cholesterol uptake has emerging role in the aggressiveness of breast cancer (Fig. 5). Overactivation of ErbB2 signaling leads to activation of the transcription factor MZF1 which in turn increases the expression of the lysosomal cholesterol transporter NPC1 and activates macropinocytosis, increasing the macropinocytotic uptake of extracellular cholesterol in breast cancer cells [7]. This is likely due to a concurrent activation of EGFR signaling [48]. Excess LDL exposure increases the cholesterol uptake and growth of triple negative as well as ER-negative breast cancer cells [60, 101] and HDL can increase the growth and migration of ER-positive cells [65, 70]. HER2 overexpressing and triple negative breast cancer xenografts implanted in LDLR-/- mice models have increased expression of LDLR when exposing the mice for excessive circulating LDL-cholesterol [59]. In these conditions the tumors grow also larger and the knockdown of LDLR in tumor cells decreases their growth. Related to this, high-cholesterol diet in BALB/c mice carrying GFP-expressing 4T1 or MDA-MB-231 breast cancer xenografts promotes intravasation of cancer cells, which can be inhibited by blocking LDL binding to LDLR [102], and human breast tumors containing higher levels of esterified cholesterol have higher LDLR expression and are more aggressive [71].

## Cholesterol and ovarian cancer

Malignant ovarian cancer ascites is rich in cholesterol [103, 104]. The most common and most aggressive form of ovarian cancer, high-grade serous ovarian cancer (HGSC) preferentially metastasizes to omentum [105], which is a visceral fat deposit mainly composed of adipocytes and being a rich source of cholesterol [106]. Accordingly, intraperitoneal injection of SKOV3ip1 ovarian cancer cells in female athymic nude mice results in their rapid accumulation to omentum [107], indicating that this fat tissue rich of triglycerides and cholesterol provides an ideal homing environment for ovarian cancer cells. Omental adipocytes and ovarian cancer cells have a mutual relationship where cancer cells can induce lipolysis in adipocytes and utilize their contents and the adipocytes on the other hand can reprogram cancer cell metabolism to promote metastasis [108, 109].

The tumor suppressor gene p53 (Tp53) mutations pay central role in the development and biology of HGSC. The loss-of-function mutations of TP53 occur in close to 100% of HGSC cases [110]. There is accumulating evidence that the wild type TP53 not only prevents tumorigenesis by regulating the transcription of genes involved in cellular proliferation, DNA repair and cellular death, but is also involved in the regulation of cholesterol metabolism, suggesting that altered cholesterol metabolism may contribute to HGSC development [111].

Functional p53 can suppress the mevalonate pathway and this occurs by p53 transcriptionally upregulating the gene expression of *ABCA1*, blocking the activation of SREPB-2, as well as a decreasing in the transcription of mevalonate pathway related genes [112]. Mutated Tp53 activates SREBP-2, and increases the expression of SOAT1, thereby increasing the expression of mevalonate pathway related genes. Activation of the mevalonate pathway will not only result in an increased cholesterol synthesis, but also in an upregulation of the expression as well as post-translational modifications of small GTPases, which will increase cellular metabolism, proliferation, and migration [111, 113, 114].

Analysis on datasets from Cancer Genome Atlas (TCGA) of the expression of LDLR and HMGCR in ovarian cancer patients, as well as a retrospective study on 65 patients found that patients with a high expression of LDLR and low expression of HMGCR in their tumors, had a poorer disease-free survival, as well as a poorer overall survival, compared to patients with a low LDLR expression [115]. High HMGCR expression correlated with a better disease prognosis. The serum LDL and cholesterol levels were significantly higher in patients that were resistant to platinum-based treatment, than in patients sensitive towards platinum-based treatment. These results suggest that the accumulation of cholesterol in cancer cells might be involved in the development of platinum-resistance of ovarian cancer, thus targeting the pathways involved in cholesterol metabolism and homeostasis could serve as an interesting therapeutic opportunities [10, 26, 115].

**Fig. 6:**
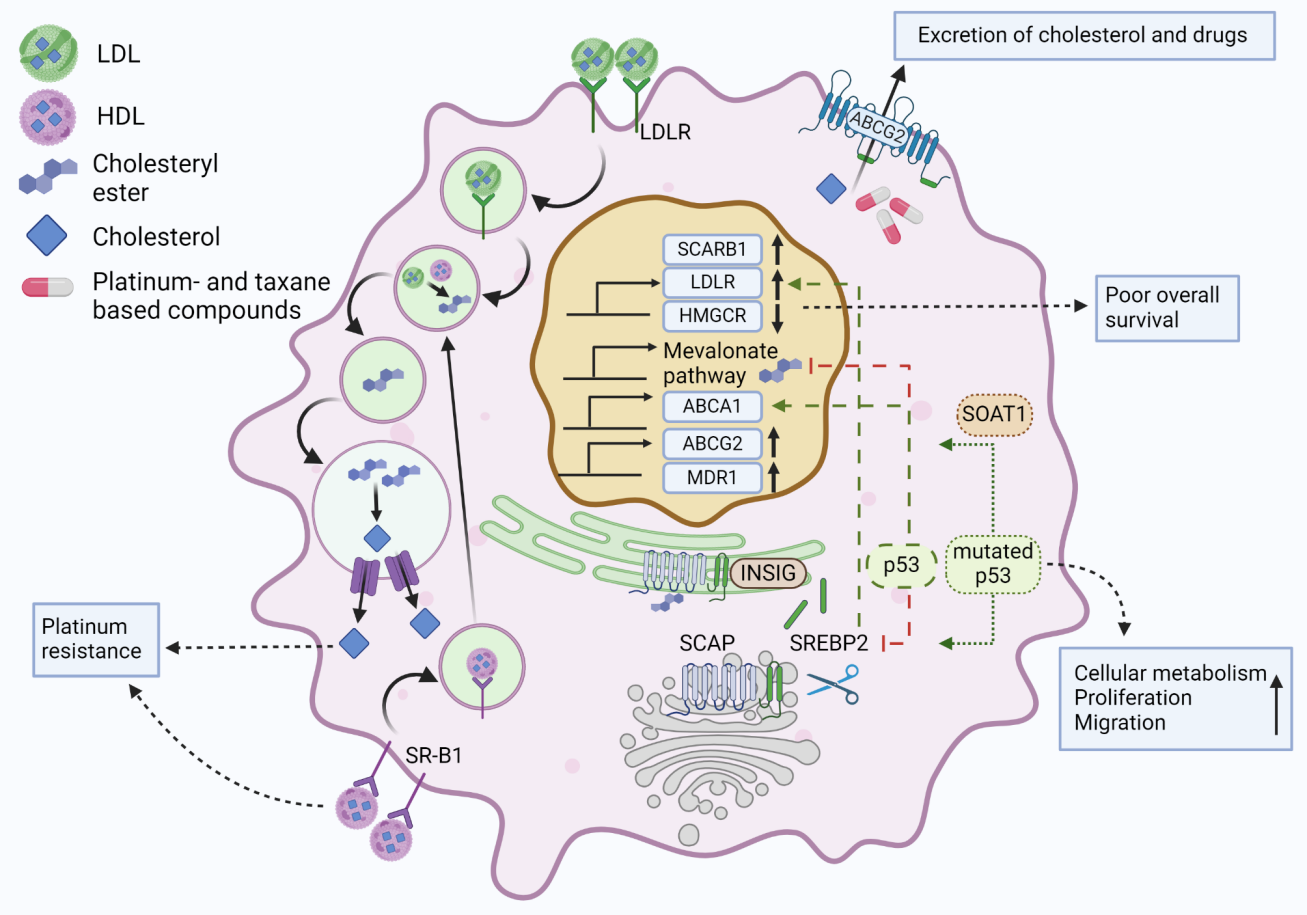
Illustration of cholesterol uptake mechanisms in ovarian cancer. The illustration highlights central molecular mechanisms that lead to altered cholesterol metabolism in ovarian cancer cells. Abbreviations: LDL (low density lipoprotein), HDL (High density lipoprotein), ABCA1 (ATP-binding cassette transporter A1), ABCG2 (ATP-binding cassette transporter G2), MDR1 (Multidrug Resistance 1), SR-B1 (Scavenger receptor B1), INSIG (Insulin-induced gene 1 protein), SOAT1 (Sterol O-acyltransferase 1), SREBP2 (Sterol regulatory element-binding protein 2), SCAP (SREBP cleavage-activating protein), HMGCR (HMG-CoA reductase)

High cholesterol content and increased ability for cholesterol uptake are connected to the aggressiveness of ovarian cancer (Fig. 6). Clinicopathological studies on ovarian cancer show clear correlation with the high cholesterol contents in ascites with chemoresistance [116]. High cholesterol content supports chemoresistance by up-regulation of ABCG2 and MDR1 which in addition to excreting excess cholesterol, can pump out platinum- and taxane-based compounds. Supportively, upon platinum resistance, high-grade serous ovarian cancer cell lines OVCAR4 and OVCAR5 exhibit higher intracellular cholesterol contents than the corresponding parental cell lines [117]. Here increased cholesterol uptake is mainly responsible for this metabolic change which is controlled by the increased SR-B1 expression caused by platinum-treatment. Cholesterol uptake inhibition by synthetic cholesterol-poor HDL-like nanoparticles inhibited cholesterol uptake and result in cell death and inhibition of murine xenograft tumor growth. Glutathione 4 peroxidase together with SR-B1 was found to control the cholesterol uptake. Another study reports of platinum-associated upregulation of LDLR in ovarian cancer cells when using cell lines SKOV3 and A2780 [118]. In this study shRNA-mediated downregulation of LDLR increased the activity of mTOR which could be overcome by inhibiting mTOR.

## Possibilities for therapies targeting cholesterol uptake in breast and ovarian cancers

Cancers often upregulate the key cholesterol uptake molecules such as NPC1, NPC1-like 1 protein (NPC1L1), LDLR, SR-B1, and proprotein convertase subtilisin/kexin like 9 (PCSK9) making them the most logical targets of possible pharmaceutical interventions. NPC1 inhibition inhibits the utilization of extracellular cholesterol by preventing its transport out from lysosomes [7, 119]. U18666A and itraconazole are direct inhibitors of NPC1. Of these two, itraconazole has most promise as a pharmaceutical invention targeting NPC1, since it is already included in several clinical trials as an anti-cancer drug due to its ability to inhibit hedgehog pathway as well as due to its various anticancer activities including its ability to target multidrug resistance via ABC transporters [120]. Currently there are 7 clinical trials registered for the use of itraconazole as a combinatory therapy in ovarian cancer patients and 6 in breast cancer patients (clinicaltrials.cov). It would be interesting as well as useful to correlate the outcome of these studies to the cholesterol uptake ability of the targeted human tumors in vivo.

Another option for direct targeting could occur via NPC1L1. Possibilities for intervention include for example the use of a drug like ezetimibe that binds to NPC1L1, and which is used in clinic on patents that are intolerable for statins [121]. Ezetimibe inhibits cholesterol absorption from the small intestine, and thus reduces circulating cholesterol levels potentially impacting tumor growth [121]. Upregulated NPC1L1 induces cholesterol uptake and increases the plasma cholesterol levels. Extracellular domain of NPC1L1 binds ezetimibe which will then block cholesterol transport [122, 123]. Higher NPC1L1 expression in late-stage ovarian tumors is connected to worse survival, suggesting that its inhibition could be beneficial [124]. On the other hand, a meta-analysis of ezetimibe suggests that its use has a slight tendency to increase the risk of breast cancer [125], indicating that more research is needed to evaluate its usability as a combinatory treatment in breast and ovarian cancers.

A recent study with platinum resistant HGSC ovarian cancer cell lines OVCAR4 and OVCAR5 shows that it is possible to decrease the uptake of cholesterol through SR-B1 with treating the cells with synthetic SR-B1-targeting, HDL-mimicking nanoparticles, and that it leads to decreased cancer cell viability [117]. Promisingly, these nanoparticles can inhibit the growth of the xenografted OVCAR5 cells in vivo. Nanoparticle targeting of LDLR is also developing [126, 127], making the future targeting of LDLR-mediated cholesterol uptake in cancer approachable. Nanoparticle targeting of cancer is developing fast and although there are still challenges with some of them in respect to their targeting to solid tumors, as well as in their sufficient accumulation, at least 10 nanomedicine candidates were already in clinical trials as cancer treatments already in 2023 [128].

Clinically usable inhibitors exist for PCSK9, which is one of the main enzymes controlling cholesterol metabolism. PCSK9 inhibitors, such as evolocumab and alirocumab which are developed to treat statin-intolerant high LDL-cholesterol patients, prevent LDLR degradation and increase LDLR recycling back to the cell membrane [129]. The main target of these inhibitors is liver, where increased LDL-cholesterol uptake via upregulation of LDLRs will decrease the circulatory levels of LDL. Despite supporting studies on PCSK9 inhibition for example as a combinatory treatment with immune checkpoint inhibition in murine 4T1 mammary cancer cell xenografts [130] and in targeting the OVCAR3 HGSC ovarian cancer cells [131] exist, generally studies with PCSK9 inhibition in cancer contain controversialities and are thus pending for more thorough studies for its usability as a cancer-treatment, since it could also induce LDLR expression in tumors, and thus increase tumor growth [132].

In terms of targeting cancer´s ability for extracellular cholesterol uptake, inhibiting cholesterol receptors LDLR and SR-B1 may not be enough, since cholesterol can also be taken up by cancer cells via macropinocytosis [7]. In epithelial cells macropinocytosis is strongly connected to oncogenic activation and actin cytoskeleton rearrangements which are generally involved in cancer progression, and for this reason inhibition of macropinocytosis as means to prevent cholesterol uptake could be a useful approach in cancers that can activate it. Interestingly, activated ErbB2-induced macropinocytosis-mediated cholesterol uptake in breast cancer cells is connected to upregulation of NPC1 and can thus be blocked by inhibiting it [7]. It remains to be seen how common is NPC1 upregulation is cancers that use macropinocytosis for cholesterol uptake, and when the first clinically approved macropinocytosis inhibitors will be developed.

## Conclusions and future directions

It is obvious that cancer cells possess and master different means to fulfil their cholesterol needs, and for this reason, all the multiple ways should be considered when planning and testing novel cancer therapies targeting cholesterol metabolism. Within this, cholesterol uptake has turned to be a significant factor in the progression of breast and ovarian cancers, and its targeting can offer promising therapeutical opportunities. Decreasing serum cholesterol levels as a strategy to support cancer treatments most commonly involves the use of drugs that inhibit mevalonate pathway, and as a result, clinical trials targeting cholesterol metabolism in cancer rely strongly on the inhibition of cholesterol synthesis [133–135]. Statins have shown some promising results as combinatory treatments in cancer, but also some non-promising ones, and are shadowed by their potential ability to induce cholesterol uptake in cancer cells. This could especially occur in well-vascularized tumors and tumors located at the vicinity of fat deposits, such as breast and ovarian tumors, raising questions about the strategy’s efficiency and inviting suggestions for its improvement. Activation of extracellular cholesterol uptake mechanisms in cancer cells and their effect on the clinical outcome presents a major unsolved issue in cholesterol metabolism and its targeting in cancer. Since various mechanisms for cholesterol uptake can be activated in breast and ovarian cancers, these should be taken into consideration when planning future studies on the efficient targeting of their cholesterol metabolism. Towards this, it would be beneficial to identify molecular signature/s that define those breast and ovarian tumors that are dependent on cholesterol uptake or can efficiently activate it upon their need. Promising novel approaches to target cholesterol uptake confer the development of the LDLR and SR-B1 targeting nanoparticles. It would be interesting to see the further development of nanoparticle targeting of LDLR and SR-B1 in preclinical studies involving for example several patient-derived breast and ovarian cancer tumor organoids with varying receptor expression levels and patterns, to support their potential usability in clinic. Another future direction could include investigation of the role of macropinocytosis in cholesterol uptake: how common it is in breast cancer and weather it is involved in the cholesterol uptake in ovarian cancer as well, what can regulate it in addition to EGFR and Ras? Are these two oncogenes determinantal for its activation, or if other means to activate actin cytoskeleton remodeling can be utilized as well? It is of course good to keep in mind that in addition to various pharmaceutical interventions, one simple way to control cholesterol uptake could be a low-cholesterol diet.

## Data Availability

Not applicable.

## Abbreviations

ABCA1: ATP-binding cassette transporter A1
ABCG1: ATP Binding Cassette Subfamily G Member 1
ACAT1/SOAT1: Acetyl-CoA Acetyltransferase
EGFR: Epidermal Growth Factor Receptor
ER: Estrogen Receptor
HDL: high density lipoprotein
HER2: Human Epidermal Growth Factor 2
HGSC: High-Grade Serous Carcinoma
HMGCR: HMG-CoA reductase
INSIG: Insulin-induced gene protein
LAMP-1: Lysosomal Associated Membrane Protein 1
LAMP-2: Lysosomal Associated Membrane Protein 2
LIMP-2/SCARBB2: Lysosomal Integral Membrane Protein 2/Scavenger Receptor Class B Member 2
LDL: low density lipoprotein
LDLR: Low density lipoprotein receptor
LXR: Liver X receptor
MAPK: Mitogen Activated Protein Kinase
mTOR: Mammalian Target of Rapamycin
MZF1: Myeloid Zinc Finger-1
NPC1 and NPC2: Niemann-Pick type C protein 1 and 2
PI3K: Phosphoinositide 3 kinases
PCSK9: Proprotein convertase subtilisin/kexin type 9
PTEN: Phosphatase and Tensin Homolog
SCAP: SREBP-2 cleavage activation protein
SR-B1: SCARBB1; Scavenger receptor class B type 1
SREBP-2: Sterol regulatory element-binding protein 2
Tp53: Tumor suppressor p53
